# Fires in Public Institutions

**Published:** 1903-06-13

**Authors:** 


### Fires in Public Institutions.

The calamitous fire at Eton, following so closely upon that at Colney Hatch, will not, we trust, be permitted to fade from the public mind without leading to some increase of precaution in connection with such outbreaks in general. The sympathy that is naturally felt for the parents of the unfortunate boys who were suffocated at Mr. Kindersley's should, if possible, be utilised while it is still active, and converted into a force by which others may be preserved from a similar fate. Apart from any special training, it is difficult to conceive a community better calculated to escape from a burning house than one consisting mainly of bold and active boys, accustomed to manly sports, and unhampered by care for the safety of persons less helpful than themselves, such as young children or the infirm; and yet, in such a community, two perished, one of them, it would appear, without being even roused to any consciousness of danger. In passing through hospitals and asylums it is quite usual to see a display of fire buckets or of fire hose; but in how many is there any definite education or practice with regard to the uses of these appliances, or any real preparation for turning them to the best account in time of need. The ascendancy or the suppression of a fire may turn upon correct action in a period of time to be reckoned in minutes. Even in private houses, it is no uncommon thing for fires to be attended by loss of life; and even where means of escape to adjoining buildings have been provided, these means have too often been out of order, or for some reason inaccessible to the inmates, when the time came at which they were required.

In the United States of America, nearly all buildings which, either from their construction, or from the uses to which they are applied, are specially exposed to fire risks are protected by an ingenious mechanical arrangement of so-called "automatic sprinklers," by which hundreds of fires have been extinguished at their very commencement. In such buildings, the ceilings of the rooms are fitted with a sufficient number of contrivances each of which resembles the rose of a watering-pot, and is in connection with the main water supply of the building, from which it is cut off by a valve held in position by a strip of "fusible metal." As soon as the temperature of the room is raised to the necessary degree, the valves open and down comes the shower. But, in a general way, brains are better than mechanism; and it is the clear duty of the authorities of every largely populated building, especially where a portion of the population consists of sick or infirm persons, to exercise sufficient foresight with regard to fire dangers. For this purpose, all the able-bodied inmates should be taught the methods of using fire appliances, and should be systematically drilled until they were expert. Each one of them should be made to learn what exact piece of duty, in the event of a fire occurring, would be required of him, and should be taught to go promptly to his proper post on the first alarm. Even private families may do something in the way of preparation for a calamity which would thus be deprived of half its terrors. The fire at Eton called forth a letter to the *Times* from "An Amateur Fireman," in which the writer showed how to escape the danger of suffocation. He makes every member of his family sleep with a pocket-handkerchief under his pillow, prepared to dip it into water and tie it wet round the mouth and nostrils as soon as the presence of smoke is recognised. Thus protected, said the writer, it is easy to walk erect and safely, where without such an appliance speedy unconsciousness would be the prelude to death.

